# Supplementary material for: Direct genome-wide identification of G-quadruplex structures by whole-genome resequencing
Source: Nat Commun. 2021 Oct 14;12:6014. doi: 10.1038/s41467-021-26312-w (PMC8516911; doi:10.1038/s41467-021-26312-w)
Supplement: Supplementary file 1 — Supplementary Information [file 41467_2021_26312_MOESM1_ESM.pdf]

Supplementary Information

Supplementary Information contains 5 figures and 9 tables.

Supplementary Figures

Supplementary Figure 1. Length distribution of PG4s.
Supplementary Figure 2. Guanine content distribution in PG4 positive segments.
Supplementary Figure 3. The influence of sequencing depth in PG4 detection.
Supplementary Figure 4. Prevalence of detected sequence by category of optimized G4-seq and G4-miner.
Supplementary Figure 5. Prevalence of detected sequence by category of rG4s and G4-miner.

Supplementary Tables

Supplementary Table 1. Information of the four known control sequences.
Supplementary Table 2. Statistic of quality drop in PG4 positive segments and PG4 negative segments.
Supplementary Table 3. Statistic of quality drop in PG4 negative segments by filtering the segments with low guanine content (G% < 28%).
Supplementary Table 4. Thresholds for the two strands of the parallel runs.
Supplementary Table 5. Sequencing coverage for two parallel runs.
Supplementary Table 6. Canonical quadruplexes detected by G4-miner.
Supplementary Table 7. MG4s in the ‘other’ category.
Supplementary Table 8. MG4s structural categories.
Supplementary Table 9. Reference genome and data source.

Supplementary Figures

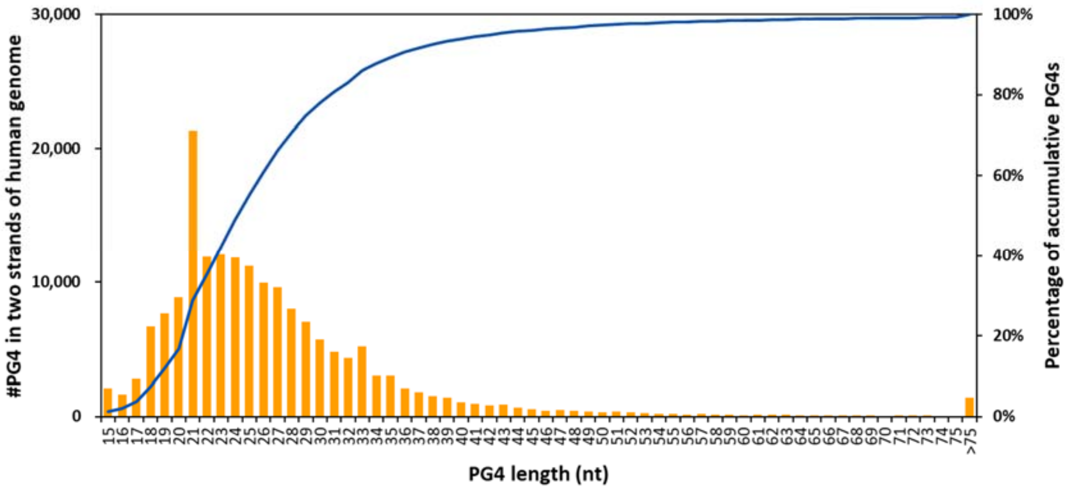

Supplementary Figure 1. Length distribution of PG4s. Over 99% PG4s are shorter than 75nt.

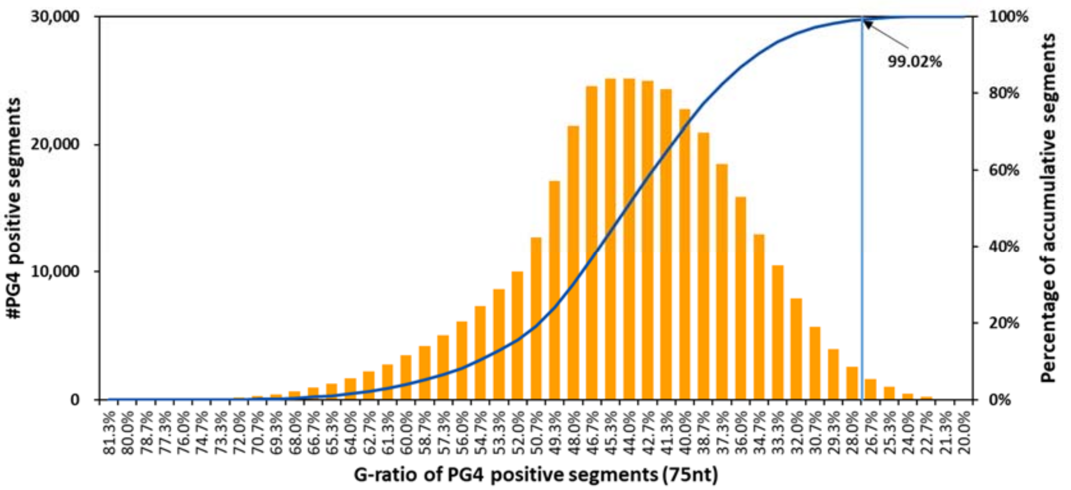

Supplementary Figure 2. Guanine content distribution in PG4 positive segments.

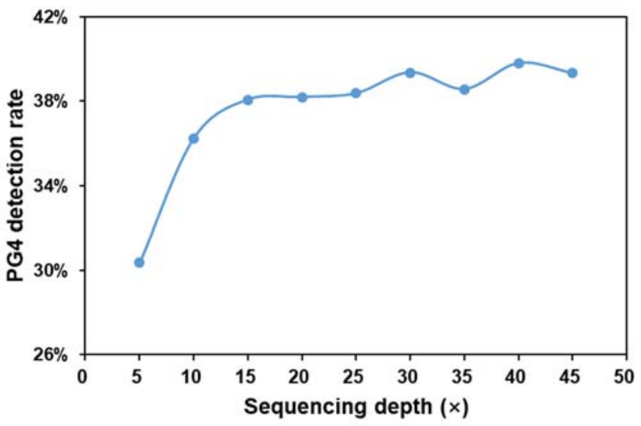

Supplementary Figure 3. The influence of sequencing depth in PG4 detection.

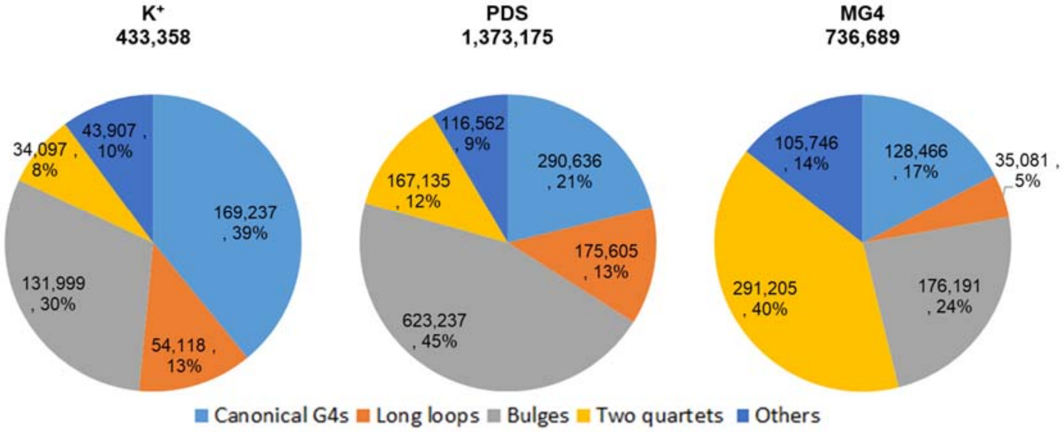

Supplementary Figure 4. Prevalence of detected sequence by category of optimized G4-seq and G4-miner. The results of optimized G4-seq generated in K^+ and K^+-PDS were reported by Marsico *et al.* in 2019 [1].

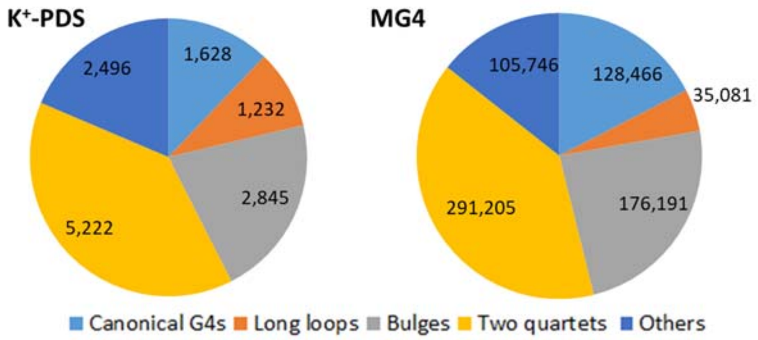

Supplementary Figure 5. Prevalence of detected sequence by category of rG4s and G4-miner. The results of rG4s generated in K^+ and K^+-PDS were reported by Kwok *et al.* in 2016 [2].

Supplementary Tables

Supplementary Table 1. Information of the four known control sequences.

Type	Name	Location	Sequence
**Positive control**	src	Chr20: 36,012,930-36,012,960	GGGTGACTTGGGTGTCCGGGGGGTGGGGGGG
	myc	Chr8: 128,748,169-128,748,190	GGGGAGGGTGGGAGAGGGTGGGG
**Negative control**	a-repeat	Chr1: 33,986,267-33,986,283	GGGTGGGAAAGGGTGGG
	g-rich	Chr1: 163,874,688-163,874,707	GGTTTTGGTTTTGGTTTTGG

Supplementary Table 2. Statistic of quality drop in PG4 positive segments and PG4 negative segments.

PG4 positive segments

		Quality score dropped											
		1	2	3	4	5	6	7	8	9	10	11	12
# loci reached threshold	1	89.16%	89.16%	87.25%	87.24%	51.61%	51.24%	51.23%	49.44%	49.40%	36.42%	32.66%	32.66%
	2	81.13%	81.12%	78.40%	78.39%	43.13%	42.62%	42.62%	41.19%	41.13%	32.25%	27.98%	27.97%
	3	74.43%	74.42%	71.29%	71.26%	39.46%	38.82%	38.82%	37.62%	37.55%	30.25%	25.72%	25.71%
	4	68.83%	68.82%	65.56%	65.52%	37.32%	36.59%	36.59%	35.48%	35.39%	28.84%	24.08%	24.07%
	5	64.12%	64.12%	60.96%	60.92%	35.73%	34.92%	34.92%	33.89%	33.79%	27.68%	22.75%	22.75%
	6	60.22%	60.22%	57.08%	57.04%	34.49%	33.60%	33.59%	32.59%	32.46%	26.67%	21.62%	21.62%
	7	56.89%	56.88%	53.94%	53.88%	33.45%	32.48%	32.47%	31.49%	31.36%	25.77%	20.63%	20.63%
	8	54.01%	54.01%	51.20%	51.14%	32.56%	31.50%	31.50%	30.54%	30.41%	24.96%	19.73%	19.72%
	9	51.53%	51.52%	48.91%	48.84%	31.75%	30.59%	30.59%	29.68%	29.54%	24.18%	18.87%	18.87%
	10	49.40%	49.40%	46.97%	46.89%	31.03%	29.80%	29.80%	28.89%	28.75%	23.48%	18.08%	18.08%
	11	47.51%	47.50%	45.26%	45.17%	30.33%	29.02%	29.02%	28.14%	27.98%	22.78%	17.33%	17.33%
	12	45.92%	45.91%	43.76%	43.66%	29.71%	28.31%	28.31%	27.41%	27.24%	22.08%	16.61%	16.61%
	13	44.45%	44.44%	42.37%	42.26%	29.10%	27.64%	27.64%	26.73%	26.54%	21.44%	15.95%	15.94%
	14	43.15%	43.14%	41.20%	41.08%	28.50%	26.97%	26.96%	26.06%	25.88%	20.78%	15.26%	15.24%
	15	41.96%	41.95%	40.16%	40.04%	27.96%	26.33%	26.32%	25.43%	25.24%	20.16%	14.61%	14.60%
	16	40.93%	40.93%	39.13%	38.98%	27.41%	25.73%	25.72%	24.82%	24.63%	19.57%	14.00%	13.99%
	17	39.90%	39.90%	38.18%	38.01%	26.87%	25.11%	25.10%	24.20%	24.00%	18.96%	13.38%	13.37%
	18	38.94%	38.93%	37.29%	37.12%	26.32%	24.48%	24.46%	23.57%	23.37%	18.36%	12.77%	12.76%
	19	38.06%	38.05%	36.44%	36.28%	25.80%	23.89%	23.88%	22.98%	22.76%	17.77%	12.19%	12.18%
	20	37.16%	37.14%	35.63%	35.45%	25.26%	23.28%	23.26%	22.34%	22.11%	17.18%	11.62%	11.61%

PG4 negative segments

		Quality score dropped											
		1	2	3	4	5	6	7	8	9	10	11	12
# loci reached threshold	1	62.75%	62.75%	59.60%	59.59%	12.83%	12.71%	12.71%	11.63%	11.61%	3.52%	3.40%	3.40%
	2	44.61%	44.61%	41.13%	41.12%	5.44%	5.30%	5.30%	4.76%	4.74%	1.76%	1.65%	1.65%
	3	33.43%	33.43%	30.14%	30.13%	3.49%	3.34%	3.34%	3.00%	2.99%	1.35%	1.25%	1.25%
	4	25.96%	25.96%	23.00%	22.99%	2.71%	2.55%	2.55%	2.31%	2.30%	1.15%	1.05%	1.05%
	5	20.67%	20.67%	18.08%	18.06%	2.28%	2.13%	2.13%	1.93%	1.92%	1.00%	0.91%	0.91%
	6	16.80%	16.80%	14.53%	14.51%	2.00%	1.85%	1.85%	1.69%	1.68%	0.89%	0.81%	0.81%
	7	13.89%	13.89%	11.93%	11.91%	1.80%	1.65%	1.65%	1.51%	1.50%	0.80%	0.73%	0.73%
	8	11.67%	11.67%	9.97%	9.95%	1.64%	1.50%	1.50%	1.37%	1.36%	0.73%	0.66%	0.66%
	9	9.95%	9.95%	8.46%	8.44%	1.51%	1.38%	1.38%	1.26%	1.25%	0.67%	0.60%	0.60%
	10	8.58%	8.58%	7.29%	7.27%	1.40%	1.27%	1.27%	1.16%	1.15%	0.61%	0.55%	0.54%
	11	7.49%	7.49%	6.36%	6.34%	1.31%	1.18%	1.18%	1.07%	1.06%	0.56%	0.50%	0.50%
	12	6.60%	6.60%	5.61%	5.59%	1.22%	1.10%	1.10%	1.00%	0.99%	0.52%	0.46%	0.46%
	13	5.87%	5.87%	4.99%	4.98%	1.14%	1.02%	1.02%	0.93%	0.92%	0.48%	0.42%	0.42%
	14	5.27%	5.27%	4.49%	4.48%	1.07%	0.96%	0.96%	0.87%	0.86%	0.44%	0.39%	0.39%
	15	4.77%	4.77%	4.07%	4.05%	1.01%	0.90%	0.90%	0.81%	0.80%	0.41%	0.36%	0.36%
	16	4.34%	4.34%	3.72%	3.70%	0.95%	0.84%	0.84%	0.76%	0.75%	0.38%	0.33%	0.33%
	17	3.98%	3.97%	3.41%	3.39%	0.89%	0.79%	0.79%	0.71%	0.71%	0.35%	0.31%	0.31%
	18	3.66%	3.66%	3.15%	3.13%	84%	0.74%	0.74%	0.67%	0.66%	0.32%	0.28%	0.28%
	19	3.39%	3.39%	2.92%	2.91%	0.79%	0.70%	0.70%	0.63%	0.62%	0.30%	0.26%	0.26%
	20	3.15%	3.15%	2.72%	2.71%	0.75%	0.66%	0.66%	0.59%	0.58%	0.28%	0.24%	0.24%

Supplementary Table 3. Statistic of quality drop in PG4 negative segments by filtering the segments with low guanine content (G% < 28%).

		Quality score dropped											
		1	2	3	4	5	6	7	8	9	10	11	12
# loci reached threshold	1	12.13%	12.13%	11.48%	11.47%	2.64%	2.59%	2.59%	2.33%	2.33%	0.79%	0.72%	0.72%
	2	8.94%	8.94%	8.16%	8.15%	1.25%	1.17%	1.17%	1.04%	1.03%	0.44%	0.37%	0.37%
	3	6.83%	6.83%	6.08%	6.07%	0.87%	0.79%	0.79%	0.70%	0.69%	0.34%	0.28%	0.28%
	4	5.38%	5.38%	4.69%	4.68%	0.71%	0.62%	0.62%	0.56%	0.55%	0.28%	0.23%	0.23%
	5	4.33%	4.33%	3.73%	3.72%	0.62%	0.53%	0.53%	0.47%	0.46%	0.24%	0.20%	0.20%
	6	3.56%	3.56%	3.03%	3.02%	0.55%	0.46%	0.46%	0.41%	0.41%	0.21%	0.17%	0.17%
	7	2.98%	2.98%	2.52%	2.51%	0.50%	0.41%	0.41%	0.37%	0.36%	0.19%	0.15%	0.15%
	8	2.54%	2.54%	2.13%	2.12%	0.45%	0.37%	0.37%	0.33%	0.33%	0.17%	0.14%	0.14%
	9	2.19%	2.19%	1.84%	1.83%	0.42%	0.34%	0.34%	0.31%	0.30%	0.16%	0.12%	0.12%
	10	1.92%	1.92%	1.61%	1.60%	0.39%	0.31%	0.31%	0.28%	0.27%	0.14%	0.11%	0.11%
	11	1.70%	1.70%	1.43%	1.42%	0.36%	0.29%	0.29%	0.26%	0.25%	0.13%	0.10%	0.10%
	12	1.52%	1.52%	1.28%	1.27%	0.34%	0.27%	0.27%	0.24%	0.23%	0.12%	0.09%	0.09%
	13	1.37%	1.37%	1.16%	1.15%	0.31%	0.25%	0.25%	0.22%	0.22%	0.11%	0.09%	0.09%
	14	1.25%	1.25%	1.06%	1.05%	0.30%	0.23%	0.23%	0.21%	0.20%	0.10%	0.08%	0.08%
	15	1.15%	1.15%	0.98%	0.97%	0.28%	0.22%	0.22%	0.19%	0.19%	0.09%	0.07%	0.07%
	16	1.06%	1.06%	0.90%	0.89%	0.26%	0.21%	0.21%	0.18%	0.18%	0.09%	0.07%	0.07%
	17	0.99%	0.99%	0.84%	0.83%	0.25%	0.19%	0.19%	0.17%	0.17%	0.08%	0.06%	0.06%
	18	0.92%	0.92%	0.79%	0.78%	0.23%	0.18%	0.18%	0.16%	0.16%	0.08%	0.06%	0.06%
	19	0.86%	0.86%	0.74%	0.73%	0.22%	0.17%	0.17%	0.15%	0.15%	0.07%	0.05%	0.05%
	20	0.81%	0.81%	0.70%	0.69%	0.21%	0.16%	0.16%	0.14%	0.14%	0.07%	0.05%	0.05%

Supplementary Table 4. Thresholds for the two strands of the parallel runs.

Sample	Strand	Quality Score dropped	Loci reached threshold	Filter threshold (G%)	Positive rate	Initial false positive rate	False positive rate after filtering
12878-1	Plus strand	4	15	28%	40.04%	4.05%	0.97%
	Minus strand	3	15	28%	44.02%	4.10%	0.84%
12878-2	Plus strand	4	12	28%	39.22%	3.41%	0.97%
	Minus strand	4	11	28%	42.56%	4.02%	0.85%

Supplementary Table 5. Sequencing coverage for two parallel runs.

Sample	Total reads	Aligned reads	Sequencing depth	Genome coverage
**12878-1**	2,138.1 M	2,117.3 M	96.2× (48.1× per strand)	98.7%
**12878-2**	1,899.5 M	1,882.2 M	85.5× (42.8× per strand)	98.8%

Supplementary Table 6. Canonical quadruplexes detected by G4-miner.

Sample	# PG4s	# MG4s	# PG4 detected in MG4	% PG4 detected in MG4	Total length of MG4s
**12878-1**	356,298	1,054,941	185,822	52.2%	182.3 M
**12878-2**		936,545	172,946	48.5%	166.2 M

Supplementary Table 7. MG4s in the 'other' category.

# Other	# Hairpin		# Poly-adenine/thymine	# i-motif	# Failed to classify
	# Triplex	# Other hairpin			
**143,853**	2,215	59,347	36,838	1,717	45,951

Supplementary Table 8. MG4s structural categories.

Species	Sequencing depth	# PG4s	# MG4s	% PG4 detected in MG4	# Canonical G4s	# Long loops	# Bulges	# Two quartets	# Others
Homo sapiens	67.8	356,298	790,108	40.81%	116,976	40,404	197,498	278,802	156,428
Mus musculus	50.3	488,391	1,172,827	57.67%	251,124	66,802	281,109	294,844	278,948
Drosophila melanogaster	81.0	10,036	57,760	42.57%	3,760	2,898	12,822	20,708	17,572
Arabidopsis Thaliana	129.0	1,203	39,599	36.99%	392	291	5,260	17,421	16,235
Caenorhabditis elegans	227.8	2,154	13,471	31.99%	631	418	2,050	4,709	5,663
Saccharomyces cerevisiae	72.5	38	2,118	44.74%	15	25	336	1,033	709

Supplementary Table 9. Reference genome and data source

Species	Reference genome source	Data source (SRR code)
Homo sapiens	https://hgdownload.soe.ucsc.edu/goldenPath/hg19/	SRR9644818
Mus musculus	https://hgdownload.soe.ucsc.edu/goldenPath/mm10/	SRR13179566
Drosophila melanogaster	https://hgdownload.soe.ucsc.edu/goldenPath/dm6/	SRR12822760
Arabidopsis Thaliana	https://ftp.ncbi.nlm.nih.gov/genomes/all/GCF/000/001/735/GCF_000001735.4_TAIR10.1/	SRR11608990
Caenorhabditis elegans	https://ftp.ncbi.nlm.nih.gov/genomes/all/GCF/000/002/985/GCF_000002985.6_WBcel235/	SRR8816429
Saccharomyces cerevisiae	https://hgdownload.soe.ucsc.edu/goldenPath/sacCer3/	SRR13747318

Supplementary References

1. Marsico, G. et al. Whole genome experimental maps of DNA G-quadruplexes in multiple species. *Nucleic Acids Res* **47**, 3862-3874 (2019).
2. Kwok, C.K., Marsico, G., Sahakyan, A.B., Chambers, V.S. & Balasubramanian, S. rG4-seq reveals widespread formation of G-quadruplex structures in the human transcriptome. *Nat Methods* **13**, 841-844 (2016).
