# Supplementary material for: Direct genome-wide identification of G-quadruplex structures by whole-genome resequencing
Source: Nat Commun. 2021 Oct 14;12:6014. doi: 10.1038/s41467-021-26312-w (PMC8516911; doi:10.1038/s41467-021-26312-w)
Supplement: Supplementary file 3 — Reporting Summary [file 41467_2021_26312_MOESM3_ESM.pdf]

nature portfolio

Corresponding author(s): Jing Tu

Last updated by author(s): Sep 16, 2021

Reporting Summary

Nature Portfolio wishes to improve the reproducibility of the work that we publish. This form provides structure for consistency and transparency in reporting. For further information on Nature Portfolio policies, see our Editorial Policies and the Editorial Policy Checklist.

Statistics

For all statistical analyses, confirm that the following items are present in the figure legend, table legend, main text, or Methods section.

n/a	Confirmed	
☒	☐	The exact sample size (*n*) for each experimental group/condition, given as a discrete number and unit of measurement
☒	☐	A statement on whether measurements were taken from distinct samples or whether the same sample was measured repeatedly
☒	☐	The statistical test(s) used AND whether they are one- or two-sided<br>*Only common tests should be described solely by name; describe more complex techniques in the Methods section.*
☒	☐	A description of all covariates tested
☒	☐	A description of any assumptions or corrections, such as tests of normality and adjustment for multiple comparisons
☒	☐	A full description of the statistical parameters including central tendency (e.g. means) or other basic estimates (e.g. regression coefficient) AND variation (e.g. standard deviation) or associated estimates of uncertainty (e.g. confidence intervals)
☒	☐	For null hypothesis testing, the test statistic (e.g. *F*, *t*, *r*) with confidence intervals, effect sizes, degrees of freedom and *P* value noted<br>*Give P values as exact values whenever suitable.*
☒	☐	For Bayesian analysis, information on the choice of priors and Markov chain Monte Carlo settings
☒	☐	For hierarchical and complex designs, identification of the appropriate level for tests and full reporting of outcomes
☒	☐	Estimates of effect sizes (e.g. Cohen's *d*, Pearson's *r*), indicating how they were calculated

Our web collection on statistics for biologists contains articles on many of the points above.

Software and code

Policy information about availability of computer code

Data collection	No software was used for data collection.
Data analysis	PG4s were predicted from hg19 using g4predict (https://github.com/mparker2/g4predict.git) according to the Quadparser algorithm by searching for the '(G{3,}[ATCG]{1,7}){3}G{3,}' pattern. Human genome sequences build 37 (hg19) downloaded from UCSC genome browser was used as the reference for mapping. All qualified sequencing reads were mapped to hg19 using the BWA-MEM (version 0.7.12-r1039) in paired-end mode. The resulted SAM files were converted into BAM format, sorted based on genome position, and indexed using SAMtools (version 1.8). The single nucleotide variant calling was performed using the HaplotypeCaller with Genome Analysis Toolkit (GATK, version 4.0.3.0). Sequencing reads were analyzed using in-house Perl scripts.

For manuscripts utilizing custom algorithms or software that are central to the research but not yet described in published literature, software must be made available to editors and reviewers. We strongly encourage code deposition in a community repository (e.g. GitHub). See the Nature Portfolio guidelines for submitting code & software for further information.

Data

Policy information about availability of data

All manuscripts must include a data availability statement. This statement should provide the following information, where applicable:

- Accession codes, unique identifiers, or web links for publicly available datasets
- A description of any restrictions on data availability
- For clinical datasets or third party data, please ensure that the statement adheres to our policy

The processed data reported in this paper have been deposited in the NCBI Gene Expression Omnibus (GEO) and are available under accession number GSE159307. Underlying sequencing data are available at the NCBI Sequence Read Archive under accession number SRP286586. The reference genomes of the 6 species are

available in the UCSC Genome Browser website (Homo sapiens, Mus musculus, Drosophila melanogaster, and Saccharomyces cerevisiae, https://genome.ucsc.edu/) or NCBI's Genome resources (Arabidopsis Thaliana, Caenorhabditis elegans, https://www.ncbi.nlm.nih.gov/home/genomes/).

Field-specific reporting

Please select the one below that is the best fit for your research. If you are not sure, read the appropriate sections before making your selection.

- [x] Life sciences
- [ ] Behavioural & social sciences
- [ ] Ecological, evolutionary & environmental sciences

For a reference copy of the document with all sections, see nature.com/documents/nr-reporting-summary-flat.pdf

Life sciences study design

All studies must disclose on these points even when the disclosure is negative.

Sample size	No sample size calculation was performed. Our study is a methodological research, and only one sample was chosen for each species.
Data exclusions	No data were excluded from the analyses.
Replication	Two parallel whole genome sequencing runs were performed to evaluated the repeatability. All attempts at replication were successful.
Randomization	Not relevant. Our study is a methodological research, and only one sample was chosen for each species.
Blinding	Not relevant. Our study is a methodological research, and only one sample was chosen for each species.

Reporting for specific materials, systems and methods

We require information from authors about some types of materials, experimental systems and methods used in many studies. Here, indicate whether each material, system or method listed is relevant to your study. If you are not sure if a list item applies to your research, read the appropriate section before selecting a response.

Materials & experimental systems

n/a	Involved in the study	
x		Antibodies
	x	Eukaryotic cell lines
x		Palaeontology and archaeology
x		Animals and other organisms
x		Human research participants
x		Clinical data
x		Dual use research of concern

Methods

n/a	Involved in the study	
x		ChIP-seq
x		Flow cytometry
x		MRI-based neuroimaging

Eukaryotic cell lines

Policy information about cell lines

Cell line source(s)	GM12878 cell line from Coriell institute was used.
Authentication	Authentication was obtained from Y-breysn Biotech. Southern blot hybridization with minisatellite (VNTR) probes was used to authenticate the cell line.
Mycoplasma contamination	The cell line was not tested for mycoplasma contamination.
Commonly misidentified lines (See ICLAC register)	No commonly misidentified cell line was used.
